# What are the perceptions of faculty and academic leaders regarding the impact of accreditation on the continuous quality improvement process of undergraduate medical education programs at Caribbean medical schools?

**DOI:** 10.1186/s12909-024-05699-2

**Published:** 2024-07-19

**Authors:** Sateesh B. Arja, Bobbie Ann Adair White, Praveen Kottathveetil, Anne Thompson

**Affiliations:** 1 Avalon University School of Medicine, Willemstad, Curaçao; 2 MGH institute of Health Professions Education, Boston, USA

**Keywords:** Accreditation, Quality Assuranace, Quality Improvement, CQI, Undergraduate Medical Education, Medical School, Regulation

## Abstract

**Background:**

Accreditation and regulation are meant for quality assurance in higher education. However, there is no guarantee that accreditation ensures quality improvement. The accreditation for Caribbean medical schools varies from island to island, and it could be mandatory or voluntary, depending on local government requirements. Caribbean medical schools recently attained accreditation status to meet the Educational Commission for Foreign Medical Graduates (ECFMG) requirements by 2024. Literature suggests that accreditation impacts ECFMG certification rates and medical schools’ educational processes. However, no such study has examined accreditation’s impact on continuous quality improvement (CQI) in medical schools. This study aims to gather the perceptions and experiences of faculty members and academic leaders regarding the impact of accreditation on CQI across Caribbean medical schools.

**Methods:**

This qualitative phenomenological study inquiries about the perceptions and experiences of faculty and academic leaders regarding accreditation’s impact on CQI. Purposive and snowball sampling techniques were used. Participants were interviewed using a semi-structured interview method. Fifteen participants were interviewed across ten Caribbean medical schools representing accredited medical schools, accreditation denied medical schools, and schools that never applied for accreditation. Interviews were audio recorded, and thematic data analysis was conducted.

**Results:**

Thematic analysis yielded six themes, including accreditation and CQI, CQI irrespective of accreditation, faculty engagement and faculty empowerment in the CQI process, collecting and sharing data, ECFMG 2024 requirements, and organizational structure of CQI.

**Conclusions:**

There is ongoing quality improvement at Caribbean medical schools, as perceived by faculty members and academic leaders. However, most of the change process is happening because of accreditation, and the quality improvement is due to external push such as accreditation rather than internal motivation. It is recommended that Caribbean medical schools promote internal quality improvement irrespective of accreditation and embrace the culture of CQI.

**Supplementary Information:**

The online version contains supplementary material available at 10.1186/s12909-024-05699-2.

## Introduction

Accreditation and regulation were created for higher education to encourage quality assurance. There is an inherent belief that accreditation ensures the quality of educational programs [1, 2]. Literature suggests that accreditation can impact medical students’ outcomes, including Educational Commission for Foreign Medical Graduates (ECFMG) certification rates [3] and performance on licensure examinations [4]. Accreditation site visits can bring curricular reforms [5] and impact educational processes at medical schools [6, 7]. However, no evidence exists that accreditation guarantees that the medical educational program meets the intended outcomes, such as better-quality physicians [1, 8].

Accreditation is a process-bound approach with predefined and published standards occurring at fixed intervals, leading to collective decisions regarding compliance [9] and, unfortunately, sometimes thought to be more of an administrative exercise than a continuous quality improvement (CQI) process [10]. Accreditation processes rely more on quantitative data [2] rather than the qualitative evaluation of the educational resources, facilities, and teaching methods provided by the programs. The emphasis is on the educational process, but the impacts on the outcomes are not evident.

Accreditation based on quality assurance (QA) focuses on minimum standards [11]. The requirement for additional markers of the impact of accreditation, such as longitudinal studies in undergraduate medical schools before and after accreditation, graduates’ performance on examinations taken later in their professions, and patient outcomes, data was emphasized [1]. The literature on the impact of accreditation on the quality of medical education has primarily focused on student outcomes, such as performance on board examinations [8]. The real ability of accreditation could lie in promoting the quality improvement (QI) process [8]. It was suggested the focus of accreditation of medical education programs should be moving from student outcomes to the QI process [8]. Accreditation aligned with the QI approach is focused on endeavoring for excellence [12]. The accreditation process across the Caribbean medical schools is more of a QA approach [6], even though strategic planning and measurable outcomes are part of the accreditation agencies’ standards in the Caribbean region. There is a need to shift the focus from quantitative data, such as student outcomes, to QI processes. QA and QI must exist while ensuring measurable outcomes for medical schools [13].

Literature suggests moving from an episodic evaluation of accreditation to a CQI process [9, 13]. The CQI should have feasible outcomes and the commitment and resources to support it [9] and should center on accreditation standards. Accreditation bodies should recommend a holistic approach to quality management and CQI rather than a listed inventory approach [14]. It was also suggested that medical school leaders should recognize and encourage the accreditation process as a drive for QI rather than as a test that must be passed [14]. Sustainable accreditation for the long-term improvement of medical education should include immediate standards (formative evaluation of the program) and long-term planning for CQI [14].

CQI in accreditation involves everyone in the organization, requires everyone to take responsibility, and views quality as the result of every single step or process [15]. External reviews by accreditation organizations may not promote this internally motivated CQI, as accreditation occurs at fixed intervals, and decisions can sometimes be penalizing. Accreditation bodies require measurable outcomes of good performance. A study across Canadian medical schools showed existing QI processes are not recognized as QI actions [16]. Medical schools should spend resources on embedding quality in the organization’s culture and improving existing QI practices [16].

In the United States, accreditation bodies like the Liaison Committee on Medical Education (LCME) made strategic planning and CQI part of accreditation standards, requiring all MD degree-granting programs to engage in CQI process. Thus, the LCME advanced the concept of formative accreditation [17]. Even accreditation bodies working in the Caribbean, such as the Caribbean Accreditation Authority for Medical Education and Other Health Professions [18] and the Accreditation Commission on Colleges of Medicine [19], have strategic planning with measurable outcomes and QA systems/CQI as one of their standards. This study aimed to explore the impact of accreditation on the CQI process at Caribbean medical schools, especially in the context of the accreditation requirements of ECFMG.

## Methods

Using a qualitative phenomenology study, we explored faculty members’ and academic leaders’ experiences and perceptions regarding the impact of accreditation on CQI processes in Caribbean medical schools. Phenomenology allows researchers to explore people’s subjective experiences [20] and understand the meaning they attribute to them, including false assumptions [21]. Thus, we used phenomenology to inquire about the subjective experiences and perceptions of faculty members and academic leaders, including associate deans and deans. Our research team consisted of four educators with varied academic backgrounds: two have expertise in qualitative research (AT and BAW), and the other two are involved in teaching undergraduate medical students (SA and PK).

### Setting and participants

The Caribbean schools included in this study are offshore medical schools and public-funded schools. The participants came from accredited Caribbean medical schools (8), schools denied accreditation (1), and schools that never applied for accreditation (1).

The sampling strategy was purposive sampling, as the authors invited participants with selected characteristics [22], such as faculty members and academic leaders who have experience with Caribbean medical education programs and the accreditation process at Caribbean medical schools. Additionally, snowball sampling was used when two additional participants were recommended by one of the participants. The principal investigator (SA) sent email invitations to 40 faculty members and academic leaders across the Caribbean medical schools. The contact information was collected from an attendee list of Caribbean medical school workshops and lists from school websites. Thirteen members indicated an interest in participating in an interview, and one of the participants recommended two additional participants.

### Data collection methods

In this phenomenological study, investigators used semi-structured interviews to gather data, exploring participants’ perspectives who had experienced educational programs in the Caribbean [23]. Semi-structured interviews use a list of questions as a framework, but participants can direct the discussion [20]. The questionnaire was developed based on the previous experience of the principal investigator conducting a study across the Caribbean medical schools regarding the impact of accreditation on Caribbean medical schools’ processes [6] and based on the questionnaire used in the study conducted by Danielle Blouin [24] with her approval. Individual interviews were chosen as most Caribbean medical schools are private and for-profit, which might hinder the faculty from freely expressing their experiences or perceptions in groups.

All interviews were recorded. The interviews were conducted using Zoom. Each interview lasted between 20 and 40 min. After completing 15 interviews, the data was sufficient in terms of quantity and data quality, as evidenced by no additional information being obtained in interviews [25].

## Appendix A: Questionnaire

## Appendix B: List of participants

### Ethical considerations

The ethical approval for this study was given by the research and ethics committee of AUSOM. The principal investigator (SA) conducted interviews at medical schools other than his. The other investigator (PK) conducted the interviews at the principal investigator’s institution to avoid power dynamics, as the principal investigator held an administrative position. The principal investigator trained PK on how to conduct interviews.

### Data analysis

All interviews were audio-recorded. All interviews were transcribed using Tactiq [26]. In this study, the criteria of credibility, confirmability, and dependability were used to ensure the strength of the data. The research team (AT, SA, BAW, & PK) reviewed the data and data analysis process. The data analysis process was carefully examined by experts with experience in qualitative research (AT & BAW). The credibility of the data was achieved using member checking [27] and data sufficiency. The text was returned to the participants to ensure that it aligned with what they had experienced for member checking [27] and validation. The authors followed the seven steps involved in the Interview Transcript Review (ITR) process, including the changes confirmed (finalized transcript) and participant amendments, changes, or comments coded by the interviewer/researcher [28, 29] to enhance the rigor of this study.

The data analysis followed the inductive approach, which involves uncovering explanations, meanings, or hypotheses from the data collected rather than assessing pre-existing theory or framework [30]. The authors used thematic analysis because it spans various theoretical and epistemological orientations and is a suitable method for understating experiences, opinions, and behaviors [31, 32].

The thematic analysis included familiarization, generating initial codes, searching for themes, reviewing, defining, naming themes, and writing the report [32]. The first step is familiarization; the principal investigator (SA) and the other investigator (PK) read and re-read the transcribed data [30]. Then, meaningful words and short sentences were specified to extract the open codes. This highlighted specific words/phrases in the data that indicate a pattern. The principal investigator (SA) gave these a preliminary code (open coding). The other investigator (PK) repeated the same independently and identified open codes. Then, we did axial coding involving merging and dividing groups [33]. When there was a disagreement between SA and PK regarding the codes, another researcher (BAW) was involved in resolving the disagreement. The principal investigator shared the codes with two other investigators (AT and BAW).

The third step was the generation of themes. It occurred from the codes in the previous step that articulate a similar expression. These codes were sorted into potential themes and identified sub-themes in this step. The fourth step was reviewing the themes; each theme was distinct and had sufficient data to support it [34]. Themes were merged if the ideas were interchangeable or removed if there was no appropriate data to support them. The fifth step was defining and naming themes. The sixth and final step was writing the report.

## Results

Fifteen participants, consisting of faculty members (8), a vice-dean (1), an associate dean (1), and deans (5) from ten different Caribbean medical schools were interviewed. Of the fifteen participants, thirteen were from accredited medical schools, one from an accreditation-denied medical school, and another from a school that never applied for accreditation. All were involved in the undergraduate medical education programs, accreditation, and self-study processes. The data analysis yielded six themes. The six themes are as follows:

1. Accreditation and CQI (3 subthemes- accreditation fosters CQI, CQI processes since the last accreditation site visit, and what can be done better to promote CQI? ).
2. CQI irrespective of accreditation (one subtheme- PDSA cycle).
3. faculty engagement and faculty empowerment in the CQI process.
4. collecting and sharing data (two subthemes- dashboards and external benchmarking).
5. ECFMG 2024 requirement, and.
6. organizational structure of CQI.

## Appendix C: Coding tree

### Theme 1: Accreditation and CQI

#### Accreditation fosters CQI

Ten participants stated that accreditation fosters CQI, as accreditation helps and promotes the CQI process. Eight of these participants were from accredited medical schools, and two of them were from non-accredited and accreditation-denied schools. They stated that medical schools start building the CQI process based on standards of accreditation, based on standards of concerns, and suggestions from the previous accreditation site visits. They also mentioned that medical schools must follow rigorous guidelines, frequent visits, and evaluations by accreditation bodies, which can help with CQI. In addition, participants stated that accreditation bodies such as CAAM-HP and ACCM in the Caribbean have incorporated strategic planning with measurable outcomes and CQI in their accreditation standards. One of the participants stated that their CQI process was informal before starting accreditation, and it improved a lot after starting the accreditation process.

*“Before 2018, there was informal quality improvement, but there is no quality control unit. Once we started the accreditation process, within the institute, we improved a lot.”* (School D, Participant 4).

*“Having those accreditation standards is important. It’s because we can only sometimes count on the educators themselves to have that same passion.”* (School H, Participant 9).

#### CQI processes since the last accreditation site visit

Most participants said that their last accreditation site visit encouraged changes. This finding was common to participants from non-accredited and accreditation-denied schools. These changes included establishing a medical curriculum committee, forming an assessment committee with assessment champions, revising institutional learning objectives, instituting changes in the governing board and organizational chart, making curricular revisions, mandating USMLE requirements for graduation, improving student support systems, and establishing the CQI committees. One participant from an accredited medical school mentioned that the physical fencing around the grounds was the only change from the last accreditation site visit.

*“The only change I noticed was when the ACCM visit was over, and we now have full fencing.”* (School F, Participant 6).

#### What can be done better to promote CQI?

Most of the participants stated that accreditation bodies could do better to promote the CQI process in the Caribbean region. An important suggestion was that the CQI process should be applied at multiple levels and at every step of a medical school’s processes.

*“What could be done to better achieve or promote CQI by accreditation at Caribbean Medical Schools is that every new group of attendees interviewed in every meeting during the accreditation process should be asked about CQI in their area of concern. From admissions to the clinical sites, continuous quality improvement should be addressed at every level of the medical school.”* (School J, Participant 12).

Participants also suggested that there should be more in-depth standards for CQI and inquiring about CQI at every step of a medical school’s processes.

The other important suggestion was for the accrediting agencies to provide better platforms for greater collaboration and knowledge sharing, facilitating the exchange of best practices and innovations, especially in the context of for-profit organizations such as Caribbean medical schools.

*“It might be beneficial to encourage greater collaboration and knowledge sharing between the Caribbean medical schools as well as to facilitate the exchange of best practices and innovations in medical education.”* (School J, Participant 11).

### Theme 2: CQI irrespective of accreditation

Most of the participants mentioned that CQI should be done regardless of accreditation. A few participants said they were making changes for accreditation and CQI purposes. The school is responsible for engaging in the CQI process for the smooth functioning of the school.

*“Continuous quality improvement is a cornerstone of excellent medical education.”* (School J, Participant 12).

*It is the school’s merit to ensure that it promotes a CQI process.* (School B, Participant 2)

As stated by the participants, CQI is a systematic way of evaluating and reviewing processes. It is data-driven and involves analyzing the current processes, finding out if there is any gap, and fixing it. One dean mentioned they do this process by researching literature and observing other schools to find the best practices. One participant mentioned that the CQI process can prepare you for any accreditation visit at any time. The majority of the participants emphasized that CQI should be implemented irrespective of accreditation to correct internal issues with robust QA and QI systems.

*“We don’t have to own that paperwork or initiate; that is already existing. It’s an ongoing process. It doesn’t matter when that accreditation is coming or not. We are almost always ready for any accreditation anytime.”* (School E, Participant 5).

Participants mentioned that CQI should begin with the leaders of the medical schools and should involve all stakeholders. Three participants (two of them are from an accredited medical school and one from a school that never applied for accreditation) commented that their schools needed to consider all stakeholders in the CQI process; they stated that they never saw important data such as USMLE pass rates and residency matching data.

*“The Deans have all the data but do not share us with all.”* (School F, Participant 6).

*“I am unaware of what happens to the students after MD5.*” (School F, Participant 7).

Two participants from an accredited medical school mentioned that nothing was done for quality improvement purposes. They had never seen a meeting of the CQI committee, and the CQI process was just for the sake of paperwork. Two other participants felt their schools need content experts for proper checks and balances.

“*They are reaching only some stakeholders. They need to take into consideration and reach out to all stakeholders.”* (School D, Participant 4).

*“What process are they maintaining for the checks and balances? But what I could sense, even from other departments, is only one or two qualified people. That’s all. So why is it such a big university maintaining that, you know, huge numbers with the minimal quality of faculty? It is highly impossible to maintain the quality of education.”* (School I, Participant 10).

#### Planning-development-study-act (PDSA) cycle

The PDSA cycle was commonly used by most institutions, according to the participants. Most reported that their schools used this or a similar process for QI. A few participants were unfamiliar with the PDSA process, but most of them had some QI process or measures in place for QI.

### Theme 3: Faculty engagement and empowerment in the CQI process

Twelve participants (eleven of them are from accredited schools and one from accreditation-denied medical school) felt that faculty are engaged and accountable to the CQI process. The change processes begin with faculty, and faculty are essential partners in this process and are required to get approval from the faculty boards. Faculty contribute different ideas and are part of the decision-making process. The key is faculty teams, part of university standing committees involved in direct governance and decision-making. Faculty are empowered as they are involved in the decision-making process and have the authority to contribute to the change process. The other way of empowering the faculty members is to train them, let them do some professional development courses, and do faculty workshops.

*“To be frank, this is a challenging task because not all the faculty are implementing changes we need. You need a lot of education for them.”* (School D, Participant 4).

### Theme 4: Collecting and sharing data (outcomes)

#### Dashboards

Caribbean schools that participated in this study use different platforms such as dashboards, websites, social media, Survey Monkey, LMS, and SharePoint to collect and share data. The various data gathered are students’ feedback (surveys), faculty feedback, NBME examination rates, residency matching data, USMLE pass rates, and graduate tracking. One of the barriers mentioned was the low response rate from students. One of the good practices mentioned by one participant is that the dean of basic sciences presents the data to all faculty members once a semester.

#### External benchmarking

It is not a common practice for Caribbean medical schools to compare their program with other schools in the Caribbean region or the USA (national averages for different data). One dean mentioned that he would depend on American schools as benchmarks, as Caribbean schools are inconsistent. One faculty member from an accredited medical school said that Caribbean medical schools have low infrastructure and a high attrition rate compared to medical schools in the USA. It was mentioned by at least one participant from the interviews that Caribbean medical schools are involved in external benchmarking informally for recruitment purposes.

### Theme 5: ECFMG 2024 requirements

Most of the participants are aware of the ECFMG 2024 requirements. However, it is surprising that some participants are unaware of the ECFMG’s announcement. A few schools are making changes for both accreditation and CQI purposes.

*“The whole gamut of accreditation is coming because 2024 is right here. Is It because of that requirement?*” (School G, Participant 8).

### Theme 6: Organizational structure of CQI

Some schools have a dedicated QI office, QA office, accreditation office, or medical education unit. Two accredited schools have dedicated deans or associate deans for quality assurance. One of the deans from a school that was denied accreditation mentioned CQI is taken care of by the curriculum committee. One of the good practices mentioned at one accredited medical school was that QI is taken care of by the assistant chair of each department. QI at some schools also includes medical student working groups.

## Discussion

The results and the impact of accreditation on CQI across the Caribbean medical schools that participated in this study are encouraging, as most participants felt that there were changes or CQI processes implemented since the last accreditation site visit. However, there is still a need for improvement, especially in embracing the culture of CQI. Sustainable accreditation should accommodate the long-term CQI process and immediate standards (formative evaluation) [14]. Accreditation helped and promoted the CQI process at Caribbean medical schools. However, participants mentioned that accreditation bodies need to elaborate the standards on CQI even though there were two standards for the strategic planning process and measurable outcomes in both ACCM and CAAM’s standards. It was recommended that CQI should be established at each level of the medical schools’ processes, starting from admissions to clinical sites, rather than CQI at the institutional level only. This can be established by elaborating accreditation standards on CQI and requiring QI processes at every level of medical education processes. Otherwise, the CQI is limited to one or two accreditation standards, and current approaches to accreditation are aligned with the QA approach. Accreditation bodies can also achieve this by creating avenues for platforms for greater collaboration and knowledge sharing, facilitating the exchange of best practices and innovations. This could be hindered due to the nature of medical schools in the Caribbean, as most of them are private-funded and for-profit organizations.

As Scrivens [15] described, CQI in accreditation involves everyone in the organization, requires everyone to take responsibility, requires leaders to support improvements, and places sound statistical analysis at the center of QI. To a certain extent, Caribbean medical schools participated in this study follow these principles. Caribbean medical schools that participated in this study, irrespective of accreditation status, are engaged in data collection and analysis, as demonstrated by using different platforms, including SharePoint, dashboards, LMS, and Survey Monkey, to collect and share data. The gathered data includes feedback from faculty and students, student pass rates in internal and external standardized examinations, residency matching data, and graduate tracking. It requires transparency and the involvement of all stakeholders. It was especially concerning that two faculty members from an accredited medical school mentioned that they never received data or were unaware of what happens to students. However, most faculty members and academic leaders mentioned that they know the data collection process and use it to analyze, evaluate, and implement the required changes.

Stakeholder satisfaction, stakeholder expectations, student and graduate performance, and engagement are indirect themes identified that accreditation could impact [24]. Seeing faculty involved in the change and decision-making processes across the Caribbean medical schools was encouraging. This is the key to stakeholder engagement in the QI process, especially faculty. There was a common consensus among faculty and academic leaders that faculty are the reason behind the ideas, initiating the change process, and approvals required by faculty boards. This was identified by each participant except two. Student surveys and feedback are also considered important stakeholders in the change process.

The ECFMG 2024 requirements can be attributed to the origin of concerns regarding accreditation in Caribbean medical schools. However, two academic leaders (one from a school that never applied for accreditation and another from an accreditation-denied medical school) who participated in this study were determined to get accreditation before 2024. A few schools are making changes and implementing the changes for both accreditation and QI purposes. One of the studies done at Canadian medical schools identified that their existing QI processes are not recognized as QI actions, and medical schools should embed the CQI in the organization’s culture [16]. This finding is similar for Caribbean medical schools. Most of the change processes at Caribbean medical schools were due to prior accreditation site visits and were based on areas of concern cited in the reports (citations) which are reactive rather than proactive. The suggestions from previous accreditation site visits fostered the change process and QI at most Caribbean medical schools. This concurs with one of the earlier studies conducted at Caribbean medical schools [6]. Irrespective of accreditation, Caribbean medical schools should be able to embed the CQI process. This requires robust internal QA and quality control units. The robust QA systems and QI committees were identified in this study at some publicly funded medical schools in the Caribbean and some accredited for-profit organizations. Some Caribbean schools still need to establish quality control units or CQI committees and depend on curriculum committees for quality improvement. If Caribbean medical schools can embrace the culture of CQI, it would be more beneficial to the medical schools. They do not need to cram through the paperwork and can be ready for accreditation anytime. The nature of correcting themselves internally rather than external push from accreditation should be the key to embracing the culture of CQI. The CQI process should be proactive rather than reactive process.

## Conclusions

As perceived by faculty members and academic leaders, there is an ongoing change process and QI at Caribbean medical schools. Most of the change processes and QI are happening because of accreditation, and accreditation is fostering QI. However, these results cannot be generalized as the sampling size was small and was a convenient sampling approach. The investigators wanted to gather the perceptions and experiences of medical educators who had experience with Caribbean medical education programs and accreditation in this region. There is wide variation among the Caribbean medical schools regarding the quality of the educational program and accreditation status. Eight schools that participated in this study are accredited (out of 15 accredited medical schools in the Caribbean), one is denied accreditation, and one has never applied for accreditation. The lack of enthusiasm to participate in this study from non-accredited medical schools, even though there are around 65 non-accredited/denied and never applied medical schools in the Caribbean, stemmed from wanting to keep their accreditation status and CQI practices private, as most are for-profit organizations. Moreover, five participants are from a single institution where the principal investigator works. However, after interviewing 15 faculty members and academic leaders at ten institutions, the authors reached data sufficiency. Qualitative studies of this nature generate hypotheses rather than proving/disproving them. Therefore, the authors recommend that Caribbean medical schools promote internal QI irrespective of accreditation and embrace the culture of CQI.

### Strengths and limitations

This study’s sampling technique’s strength was that the subjective experiences of faculty and academic leaders were inquired, and they had gone through the accreditation process and had experiences with the Caribbean medical education programs. Another notable strength lies in the diverse array of perspectives on the accreditation process, encompassing input from various faculty members and administrators, each with unique viewpoints. Moreover, this study included a mix of schools differing in size and organizational structures. However, it’s essential to acknowledge a limitation in the sampling strategy, as it relied on a convenience sample approach. The sampling technique’s weakness in this study was that it gathered perceptions of faculty members and academic leaders at Caribbean medical schools without external input. However, the rigorousness of this study was achieved as interviewers interviewed 15 faculty members and academic leaders at multiple institutions representing ten Caribbean medical schools.

### Validity and reflexivity

The principal investigator always felt that accreditation would impact the CQI at undergraduate medical schools. He believed that the accreditation process should have impacted or promoted the CQI processes in undergraduate medical education programs. As we were going through this research project, there was a risk of inducting his bias into this research study. To avoid this, another investigator independently did the coding process and data analysis along with the principal investigator. Two other investigators supervised the entire process. The principal investigator (SA) initially performed open coding. Another investigator (PK) independently repeated this process and identified open codes. In cases of disagreement between SA and PK regarding the codes, a third researcher (BAW) mediated to resolve the differences. The principal investigator then shared the codes with two additional investigators (AT and BAW). Experts in qualitative research (AT and BAW) meticulously reviewed the data analysis process. To ensure the credibility of the data, member checking [27] and data sufficiency were employed. The text was returned to the participants for member checking [27] and validation to confirm that it accurately reflected their experiences.

### Electronic supplementary material

Below is the link to the electronic supplementary material.

Supplementary Material 1

Supplementary Material 2

Supplementary Material 3

## Data Availability

The coding tree derived from the transcribed texts is submitted as supplementary materials under Appendix C. The datasets generated and/or analyzed during the current study, such as transcribed texts, are not publicly available due to the identifiable information of the institutions that participated in this study, even though no personal information of the participants was identified. However, datasets are available from the corresponding author upon reasonable request.
