# Supplementary material for: What are the perceptions of faculty and academic leaders regarding the impact of accreditation on the continuous quality improvement process of undergraduate medical education programs at Caribbean medical schools?
Source: BMC Med Educ. 2024 Jul 19;24:781. doi: 10.1186/s12909-024-05699-2 (PMC11264946; doi:10.1186/s12909-024-05699-2)
Supplement: Supplementary file 1 — Supplementary Material 1 [file 12909_2024_5699_MOESM1_ESM.pdf]

Appendix A- Questionnaire

The questionnaire as below

1. When was your institution last evaluated for accreditation? How involved were you in this process?
2. What did you personally do during your institution's most recent accreditation process?
3. How is your institution responding to the new ECFMG requirements?
4. To what extent do you believe that the Caribbean system of accreditation (CAAM-HP / ACCM) promotes CQI process in undergraduate medical education programs? What could be done to better achieve or promote CQI by accreditation at Caribbean medical schools?
5. The accreditation cycle is three-six years, so your undergraduate medical education program has had at least one accreditation visit over the last three-six years. Reflecting on the last three-six years, what processes have been put in place in your school to fulfill the requirements of quality assurance and quality improvement?
6. How does your undergraduate medical education program use the following tools regarding accreditation?

   a. Formal CQI processes such as the plan-do-study-act (PDSA)cycle?

   b. Dedicated teams of employees for quality / process improvement?

   c. Systematic collection of program performance indicators?

   d. Student satisfaction measures elicited through surveys etc.?

   e. Iterative process where data is fed back to managers or leaders, and reviewed again later?

f. Competitive benchmarking against other programs?

g. Empowerment of employees to effect changes?]

The questionnaire was modified based on the questionnaire developed by Blouin (2020) study.
