# Supplementary material for: What are the perceptions of faculty and academic leaders regarding the impact of accreditation on the continuous quality improvement process of undergraduate medical education programs at Caribbean medical schools?
Source: BMC Med Educ. 2024 Jul 19;24:781. doi: 10.1186/s12909-024-05699-2 (PMC11264946; doi:10.1186/s12909-024-05699-2)
Supplement: Supplementary file 2 — Supplementary Material 2 [file 12909_2024_5699_MOESM2_ESM.pdf]

Appendix B- List of Participants

Number	Name of the school	Name of the participant	Position	Accreditation status
1	School A	Participant 1	Vice-dean<br>The lead of the self-study committee and accreditation site visit for his school.	Accredited
2	School B	Participant 2	Dean<br>The lead of the self-study committee and accreditation site visit for her school.	Accredited
3	School C	Participant 3	Lecturer in Internal Medicine<br>Involved in self-study and assessment committee.<br>Involved in accreditation meetings during the site visit.	Accredited
4	School D	Participant 4	Dean of Academics/Dean of Basic Sciences<br>Involved in self-study of the school.	In the process of submitting application/never applied so far
5	School E	Participant 5	Course director of the Clinical Skills department.<br>Involved in self-study and accreditation meetings during the site visit.	Accredited
6	School F	Participant 6	Course director for clinical skills.<br>Chair of the	Accredited

			promotions committee. Involved in self-study and accreditation meetings during the site visit.	
7	School F	Participant 7	Faculty of Clinical Skills department Involved in accreditation meetings during the site visit.	Accredited
8	School G	Participant 8	Associate Professor of Neuroscience Involved in accreditation meetings during the site visit and self-study.	Accredited
9	School H	Participant 9	Dean of the School of Medicine.<br>The lead of the self-study committee.<br>Chair of the curriculum committee and faculty senate.	Accreditation denied
10	School I	Participant 10	Associate Professor of Pathology.	Accredited
11	School J	Participant 11	Dean of Basic Sciences, Professor of Pharmacology.<br>Involved in self-study, accreditation meetings during the site visit, and chair of the CQI committee.	Accredited

12	School J	Participant 12	Dean of Clinical Sciences<br>Chair OBGYN department<br>Involved in self-study, accreditation meetings during the site visit, and chair of the faculty senate.	Accredited
13	School J	Participant 13	Associate dean of admissions.<br>Involved in self-study, accreditation meetings during the site visit, and chair of the admissions committee.	Accredited
14	School J	Participant 14	Chair of the promotions committee<br>Professor of Pathology.<br>Involved in accreditation meetings during the site visit.	Accredited
15	School J	Participant 15	Associate Professor of Microbiology & Immunology<br>Involved in accreditation meetings during the site visit.	Accredited
