# Supplementary material for: What are the perceptions of faculty and academic leaders regarding the impact of accreditation on the continuous quality improvement process of undergraduate medical education programs at Caribbean medical schools?
Source: BMC Med Educ. 2024 Jul 19;24:781. doi: 10.1186/s12909-024-05699-2 (PMC11264946; doi:10.1186/s12909-024-05699-2)
Supplement: Supplementary file 3 — Supplementary Material 3 [file 12909_2024_5699_MOESM3_ESM.pdf]

Appendix C- Coding tree

Codes	Categories/subthemes	Themes
Informal QI before accreditation	Accreditation and continuous quality improvement (CQI) are inseparable.	Accreditation and continuous quality improvement (CQI)
Can't separate		
Started accreditation, improved a lot		
Accreditation help and promotes CQI		
CQI inspired by accreditation		
Improve on the standards of concern and suggestions		
Important to have these standards		
Only sometimes can count on educators		
Aware of the CQI process due to accreditation process		
Guidelines for evaluation		
Frequent visits and monitoring		
Accreditation bodies recommending CQI		
introduced two standards related to CQI		
Go through rigorous accreditation process		
Address CQI at multiple levels.	Accreditation bodies can do better for CQI	
IS-2 of CAAM related strategic planning & planning process not CQI		
More in-depth standards		
Inquiring about CQI		
Greater collaboration and knowledge sharing.		
Facilitate the exchange of best practices and innovations		
Mandatory CQI		

Random visits		
CAAM-HP was initially weaponized		
Meet faculty or departments individually		
May reveal the truth		
Prepared/memorizing answers		
For the sake of formality		
Cross-campus collaboration	CQI processes in the last three-six years after the last accreditation site visit.	
Institutional learning outcomes		
Medical Curriculum Committee		
Assessment committee		
Assessment champions		
Student support systems		
Established, CQI committee and the dean for quality assurance (QA), accreditation, and strategic planning		
Proper simulation lab		
Curricular revision		
Clinical tutor fellowship program		
Some changes are coming		
Governing board, organizational chart		
NBME subject examinations		
USMLE Steps 1 and 2 & OSCE for graduation		
Boot camps for students		
Team-based activities		
Code of conduct policy		
Library assisting students		
More streamlined meetings		
Fencing around campus		
Merit of the school to promote CQI		CQI irrespective of accreditation

Cornerstone of excellent medical education		
A culture of correcting		
Robust quality assurance/quality control		
Robust CQI for smooth running		
Automatically meets the requirements		
Always ready for any accreditation		
Begins with the leaders		
Interacts with all stakeholders		
Comes naturally.		
Include all stakeholders		
Systematic way of evaluating		
Reviewing the processes & data driven		
Watch literature and other schools		
Current standing, gap, plan of action to fix that		
Student feedback		
Change internally		
Self-study process		
Collaborative feedback to improve		
Submit recommendations to curriculum committee		
Not doing anything		
Sake of paperwork.		
Never seen anything or any meeting with CQI committee.		
Require more qualified people and content experts		
Implementation and evaluation are the problems		
Not aware of it	Plan-do-study act cycle (PDSA)	
Not labeled PDSA		
PDSA better word		

PDSA-to identify the areas of improvement		
Senior Associate dean for PDSA		
Faculty board's approval		Faculty engagement and faculty empowerment in the CQI process
Contribute different ideas and decision-making.		
Take care of changes		
Equally important, and partners.		
Faculty as a team		
Changes begin with faculty		
Involve in the process		
To reach and considerate all stakeholders		
Brainstorming faculty		
Goes through the content experts, good checks, and balances.		
Empower every member		
Train them		
Empowerment by courses		
Faculty meetings and faculty workshops		
Attending conferences		
Setting up committees		
Proper hierarchy		
Student government		
Student association President and vice-president attend board meetings		
Culture of collaboration		
Not aware of what happens to students		
Don't share with faculty		
No proper mentoring		
Never took care of what faculty are suggesting		
Could not say anything		

Data feeds to the cross-campus	Dashboards, Website, social media, Survey Monkey, LMS, Share Point	Collecting and Sharing data (Outcomes)
Both quantitative and qualitative data		
Internal examinations		
USMLEs and NBME examination		
Faculty feedback		
Student feedback-surveys		
Alumni and Graduate tracking		
Peer-evaluation		
Data available to all		
Dean of basic sciences presents data		
Not a comprehensive survey		
Not aware of what happens to students		
Don't share with faculty		
By marketing team	External benchmarking with Caribbean and USA medical schools	
Benchmark NBME reports		
USA schools		
Caribbean schools-not consistent		
Observing American schools & Caribbean schools.		
Higher student attrition and low infrastructure		
The whole gamut of accreditation came because of 2024 ECFMG requirement.		ECFMG 2024 requirement
ECFMG requirement became part of the drive		
Administration informed faculty		
Hope to have this well before 2024		
Accreditation for both ECFMG requirements and for CQI processes.		

School decided to go for accreditation		
Don't know.		
Dedicated QI office		Organizational structure of CQI
Assistant chairs of the department		
Medical education department		
Consultant		
Curriculum committee		
Quality control unit headed by Vice-chancellor		
QUAC		
Different QA units		
CQI committee for each campus		
Self-study committee-QA committee, administrators and students		
Medical student working group		
Various persons working on self-study		
Dedicated teams for CQI		
Addition to regular jobs		
